# Integrated Whole Transcriptome Profiling and Bioinformatics Analysis for Revealing Regulatory Pathways Associated With Quercetin-Induced Apoptosis in HCT-116 Cells

**DOI:** 10.3389/fphar.2019.00798

**Published:** 2019-07-17

**Authors:** Zheyu Zhang, Bin Li, Panpan Xu, Bo Yang

**Affiliations:** ^1^Department of Gastroenterology, Xiangya Hospital, Central South University, Changsha, China; ^2^Department of Gastroenterology, Affiliated Hospital of Guilin Medical University, Guilin, China; ^3^Department of Integrated Traditional Chinese & Western Medicine, The Second Xiangya Hospital, Central South University, Changsha, China; ^4^Department of Integrated Traditional Chinese & Western Medicine, Xiangya Hospital, Central South University, Changsha, China

**Keywords:** quercetin, colorectal cancer, coding and non-coding RNA, transcriptomic analysis, network analysis

## Abstract

Quercetin (QUE) is a bioactive component that belongs to the natural flavonoids group, and recent researchers found that it could prevent colorectal cancer (CRC). However, the exact mechanism by which QUE exerts its anti-tumor effects in CRC remains unclear. In this study, MTS assay and flow cytometry were used to detect the anti-tumor effects of QUE on HCT-116 cells. The results showed that QUE could inhibit the proliferation and induce apoptosis of HCT-116 cells. Furthermore, whole transcriptome sequencing was employed to establish the microRNA (miRNA), long non-coding RNA (lncRNA), circular RNA (circRNA), and mRNA profiles. A total of 240 differentially expressed lncRNAs (DElncRNAs), 131 circRNAs (DEcircRNAs), 83 miRNAs (DEmiRNAs), and 1415 mRNAs (DEmRNAs) were identified in the QUE-treated HCT-116 cells compared to the untreated HCT-116 cells. Then, quantitative real-time polymerase chain reaction (qRT-PCR) was used to validate the expression of selected circRNAs, miRNAs, lncRNAs, and mRNAs. Gene Ontology (GO) and Kyoto Encyclopedia of Genes and Genomes (KEGG) analysis were performed to further investigate RNAs’ biological functions and potential mechanisms. Based on the theory of competing endogenous RNA (ceRNA), the circRNA–miRNA–mRNA and lncRNA–miRNA–mRNA regulatory networks were constructed to illustrate the regulatory relationship between non-coding RNA (ncRNA) and mRNA. Our results provided novel information about the molecular basis of QUE in treating CRC. Our findings indicated that deep RNA sequencing analysis of mRNA and ncRNAs was a promising approach to research anticancer mechanisms.

## Introduction

Colorectal cancer (CRC) is one of the most prevalent malignancies, ranking as the second leading cause of cancer-induced death worldwide (Bray et al., 2018). According to global statistics, it is estimated that CRC disease will affect more than 2.2 million new patients and will cause 1.1 million cancer deaths by 2030 (Arnold et al., 2017). In the United States, the number of new cases of CRC reached 145,600, and the number of deaths reached 51,020 in 2019 (Siegel et al., 2019). In recent years, despite advances in diagnosis and treatment of CRC, any of the current therapies cannot offer an effective therapeutic outcome due to the poor prognosis and high recurrence rate. The mortality rates are still high and unacceptable (Hamilton, 2018; Peng et al., 2018). Therefore, there is an urgent need for new CRC therapies.

Quercetin (QUE), a bioactive component belonging to the natural flavonoids group, is distributed in fresh onions, fruits, and vegetables (Russo et al., 2014). Previous studies suggested a positive association between QUE intake and improved outcomes of several types of cancer treatment (Hashemzaei et al., 2017). Recently, intense attention has been paid to the chemo-preventive and anti-tumor functions of QUE on colon cancer and increasing experimental evidence verified these effects *in vivo* and *in vitro*. Moreover, extensive studies had reported that QUE has anti-cell proliferation, pro-apoptosis, anti-angiogenesis, and anti-metastasis effects on colon cancer (Darband et al., 2018). In addition, Maria et al. confirmed that the CB1 receptor mediated the anti-proliferative and pro-apoptotic of QUE effects in human colon cancer cell lines (Refolo et al., 2015). A transcriptomics and proteomics research was conducted to characterize gene and protein changes occurring in the distal colon mucosa of rats supplemented with QUE; this further enriches the anti-cancer mechanism of QUE *in vitro* (Dihal et al., 2008). Although multiple lines of studies demonstrated the efficacy of QUE, the exact mechanism of its anti-tumor effects in CRC remains unclear. Hence, more advanced tools with large-scale data are required for the deep interpretation of the problems.

Non-coding RNAs (ncRNAs) are an abundant class of RNAs that typically do not encode proteins but functionally regulate protein expression (Mattick and Makunin, 2006). As part of RNA–protein complexes in regulating gene expression, ncRNAs can be subdivided into several families based on their size and biogenesis pathways, including microRNAs (miRNAs, with <200 nucleotides), long ncRNAs (lncRNAs, with a length >200 bp), and circular RNAs (circRNAs, with a closed continuous loop) (Cech and Steitz, 2014; Thum, 2014; Thum and Condorelli, 2015). These different ncRNAs are further classified based on sequence or structure conservation, subcellular localization and function, and association with annotated protein-coding genes and other DNA elements of known function (St Laurent, 2015). Accumulating evidence indicated that ncRNAs are involved in a remarkable variety of biological functions. Moreover, multiple lines of evidence have linked ncRNA mutations and dysregulation with various human diseases, especially cancers. Colon cancer is characterized by genetic and epigenetic modifications; thus, ncRNAs were emerging key regulators of gene expression under colon cancer (Hu et al., 2014; Guo, 2016; Zhou et al., 2016; Li et al., 2018). Hamfjord et al. (2012) profiled at least 37 differentially expressed miRNAs between CRC tissues and normal colon mucosa. Hofsli et al. (2013) detected that 21 miRNAs differentially expressed in serum of CRC patients may be applied to identify colon cancer patients at an early stage of the disease. The expression profile of circRNA in CRC tissues has also been identified, which could be used as new biomarkers for prognosis and diagnosis of CRC (Bachmayr-Heyda et al., 2015). A recent report has summarized that the lncRNA regulating cancer cell proliferation, migration, and apoptosis might also serve as biomarkers for CRC diagnosis and prognosis, including LOC285194, RP11-462C24.1, BANCR, NR_034119, NR_029373, lncRNA-AFAP1-AS1, and NR_026817.79 (Deng et al., 2017).

However, few of transcriptomes concerned about the transcriptomic effect of QUE intervention against CRC were published, and various questions remain unanswered with respect to the ncRNAs in response to QUE treatment. Thus, the whole transcriptome sequencing technique was used to profile the coding transcriptome and ncRNAs changes occurred in CRC cell response to QUE treatment. Additionally, Gene Ontology (GO) and Kyoto Encyclopedia of Genes and Genomes (KEGG) analyses were conducted to explore the biological roles and potential signaling pathways of these differentially expressed mRNA and ncRNAs. Moreover, co-expression networks were constructed based on these sequencing data and bioinformatics analysis. These results could provide novel insights into the molecular basis of QUE in treating CRC.

## Materials and Methods

### Cell Lines and Cell Culture

The human colon cancer cell line (HCT-116) was purchased from Shanghai Institutes for Biological Sciences (SIBS, Shanghai, China). The cells were cultured in Dulbecco’s modified Eagle’s medium contained in tissue culture flasks. The medium was supplemented with 10% fetal bovine serum (FBS), antibiotics, and 1% glutamine. The cells were cultured at 37°C under a humidified atmosphere containing 5% CO_2_ and 95% air.

### Cell Viability and Proliferation Assay

HCT-116 cells were seeded in 96-well plates and intervened with various concentrations of QUE (100 μΜ, 150 μΜ, 200 μΜ, and 250 μΜ) for 24, 48, and 72 h. Following incubation, cell viability was measured by the 3-(4,5-dimethylthiazol-2-yl)-5-(3-carboxymethoxyphenyl)-2-(4-sulfophenyl)-2H-tetrazolium (MTS) assay (Promega Corporation, Madison, USA, 0000328878). IC_50_ values were calculated by interpolation from dose–response curves.

### Cell Apoptosis Detection

In brief, HCT-116 cells were seeded in six-well plates at a density of 4 × 10^4^/well and incubated with QUE at different concentrations (100, 150, and 200 μΜ) for 72 h. Then, cells were collected by centrifugation, washed with phosphate-buffered saline, and resuspended with 400 µl of buffer. After that, cell suspensions were stained with Annexin V-FITC and propidium iodide (PI) according to the manufacturer’s instruction. At last, cell apoptosis detection was performed by a flow cytometer (Shanghai Pudi Biotechnology Co., Ltd., BDFACS Calibur) and analyzed by FlowJo 7.6.

### RNA Extraction and Quality Monitoring

Transcriptomic analysis was performed on untreated HCT-116 cells and HCT-116 cells treated with 150 μΜ QUE for 48 h. At first, total RNA of HCT-116 cells was extracted from three independent experiments by Trizol reagent (Invitrogen, Carlsbad, CA, USA) in accordance with the manufacturer’s protocol. RNA quantity and quality were measured by a spectrophotometer (Thermo Fisher Scientific, Wilmington, DE, USA) and the Agilent 2100 Bioanalyzer (Agilent Technologies).

### RNA Library Construction, Quality Control, and Sequencing

The small RNA library (sRNA library) was constructed according to the manufacturer’s instruction of TrueSeq small RNA library prep kit (Illumina San Diego CA, USA), and 2.5 ng RNA per sample was used as the initial amount. The T4 RNA Ligase 1 was ligated to the 3′ end of the RNA, followed by the ligation of T4 RNA Ligase 2 (truncated) to the 5′ adapter. After that, the RNA was reverse transcribed to synthesize cDNA. Finally, small RNA libraries were obtained by screening these recovered fragments using gel separation technology. In the construction of lncRNA library (chain-specific library for removal of ribosomal RNA), the epicenter Ribo-ZeroTM kit was used to remove the ribosomal RNA. Then, rRNA-depleted RNA was fragmented and used as a template to construct the cDNA library. The qualities of the libraries were further tested following these steps: 1) Initial quantification was performed using Qubit 2.0, and the insert size of the library was tested using Agilent 2100. 2) The Q-PCR method was used to accurately quantify the effective concentration of the library (effective library concentration > 2 nM). After the libraries’ quality tests were passed, different libraries performed pooling according to the amount of target data, followed by sequencing on the Illumina HiSeq platform.

### Data Processing

Based on Sequencing-By-Synthesis (SBS) technology, the Illumina HiSeq high-throughput sequencing platform sequenced cDNA libraries to produce a large amount of high-quality data, i.e., raw data. In order to ensure the accuracy of information analysis, quality control of the original data is required to obtain a high-quality sequence, which is clean reads. The exclusion criteria for quality control of the original sequence measured by the deribosomal library included the following: 1) low-quality data, 2) reads containing jointed-sequence, and 3) reads containing N (undetermined base information) ratio greater than 5%. The exclusion criteria for quality control of the original sequence measured by the sRNA library included the following: 1) low-quality data, 2) reads containing N ratio greater than 10%, 3) reads without 3′ linker sequence, and 4) sequences shorter than 15 or longer than 35 nucleotides.

The targets for miRNAs were predicted using miRanda (Betel et al., 2008) and targetscan (Lewis et al., 2003). The targets for lncRNAs were predicted based on position relationship (within 100 kb of lncRNA) and Pearson correlation coefficient (when sample size >5, the absolute value of correlation >0.9, and *p* value < 0.01) between lncRNA and mRNA. The source genes for circRNAs were acquired according to the position of the circRNA sequence; namely, the gene on which the circRNA sequence was located was deemed as its source gene.

### Quantitative Real-Time PCR Validation

To validate the expression levels of the selected lncRNAs, miRNAs, circRNAs, and mRNAs by quantitative real-time polymerase chain reaction (qRT-PCR), RNA samples from the untreated HCT-116 cells and HCT-116 cells treated with 150 μΜ QUE for 48 h were collected. Total RNA was isolated by using the Trizol reagent (Invitrogen, USA), and then reverse-transcribed into cDNA using SuperScript III Reverse Transcriptase (Invitrogen, USA) according to the manufacturer’s instruction. A Gene Amp PCR System 9700 (Applied Biosystems, USA) and 2× PCR Master Mix (Arraystar, USA) were used to perform qRT-PCR in accordance with the manufacturer’s instructions. The circRNA, lncRNA, and mRNA expression levels were normalized to β-actin (Human), an endogenous reference transcript, and calculated using the standard curve-based method (Larionov et al., 2005). The expression levels of the selected miRNAs, being normalized against the U6 (human), were calculated by the 2^−ΔΔCt^ method using untreated control as the calibrator (Livak and Schmittgen, 2001). The sequences of all primers are shown in **Table 1**. The data represent the means of three experiments.

**Table 1:** Primers designed for qRT-PCR validation of candidate miRNAs, mRNAs, circRNAs and lncRNAs.

	Forward primer	Reverse Primer	Tm (°C)	Product length
β-actin	5′GTGGCCGAGGACTTTGATTG3′	5′CCTGTAACAACGCATCTCATATT3′	60	73
U6	5′GCTTCGGCAGCACATATACTAAAAT3′	5′CGCTTCACGAATTTGCGTGTCAT3′	60	89
hsa-miR-125b-2-3p	5′GGGGTCACAAGTCAGGCTCT3′	5′GTGCGTGTCGTGGAGTCG3′	60	64
hsa-miR-338-3p	5′GGGGGTCCAGCATCAGTGA3′	5′GTGCGTGTCGTGGAGTCG3′	60	65
hsa-miR-320b	5′AGCTGGGTTGAGAGGGCAA3′	5′GTGCGTGTCGTGGAGTCG3′	60	57
hsa-miR-5096	5′GGGGAGTTTCACCATGTTG3′	5′CAGTGCGTGTCGTGGAGTC3′	60	66
LRG1	5′GGAGCAGACAGCGACCAAA3′	5′CAGCAGCAGCAGGAACAGAG3′	60	74
AZGP1	5′GGAGACCCTGAAAGACATCGTG3′	5′AACCAAACCTTCCCTGCAATAC3′	60	74
TRIM29	5′ATGCGCCACGTTGAGAAGAT3′	5′CGAGGGCTGGTATGATGTCC3′	60	204
APOBEC3G	5′CATCGTGACCAGGAGTATGAGG3′	5′AAGTAGTAGAGGCGGGCAACA3′	60	137
2:206841107|206881891	5′GAGCAAGCAGATGAGGCAAAG3′	5′GGTGATGTGACAATCCAGGTAGC3′	60	126
2:206866697|206881891	5′AGGCAAAGCACACTCTGAAGC3′	5′GGGAACATCTGGCAGGTCTAA3′	60	116
ENST00000313807	5′TCAAATCTCCCTGGGTCTCC3′	5′TATCCAAAGGCATCGTCTATCT3′	60	217
MSTRG.128888.1	5′CAAAGGTCACAGACATTCAGGCA3′	5′CCAGTTCTTACACCTTCGGTCATC3′	60	242

### GO and KEGG Pathway Analysis

To better understand the biological functions and potential mechanisms of ncRNAs and mRNAs in the mechanism of QUE acting on CRC, we applied GO enrichment and KEGG pathway analyses on these differentially expressed mRNAs (DEmRNAs), source genes of differentially expressed circRNAs (DEcircRNAs), predicted target genes of differentially expressed miRNAs (DEmiRNAs), and predicted target genes of differentially expressed lncRNAs (DElncRNAs). Briefly, GO analyses (www.geneontology.org) consisted of three components: biological process (BP), cellular component (CC), and molecular function (MF). KEGG analyses were carried to investigate the potential significant pathways (http://www.genome.jp/kegg/).

### Construction of Co-expression Network

CircRNA, lncRNA, and mRNA could inhibit target gene regulation of miRNA to indirectly regulate gene expression, serving as a miRNA sponge to bind miRNA competitively with its binding sites, which was called competing endogenous RNA (ceRNA). Based on the theory of ceRNA, the circRNA–miRNA–mRNA and lncRNA–miRNA–mRNA network were constructed and visually displayed using the Cystoscope software V3.5.0 (San Diego, CA, USA), as previous described. Different shapes and colors represent different RNA types and regulated relationships, respectively.

### Statistical Analysis

Statistical differences were determined by using SPSS 22.0 software. A *p* value less than 0.05 was considered to be significant. For sequencing data, we analyzed DE ncRNAs and DEmRNAs using DEseq (Anders and Huber, 2010) software, with *p* < 0.05 and |log2 (fold change)| > 2 as screening criteria.

## Results

### QUE Inhibits the Proliferation of HCT116 Cells

**Figure 1:**
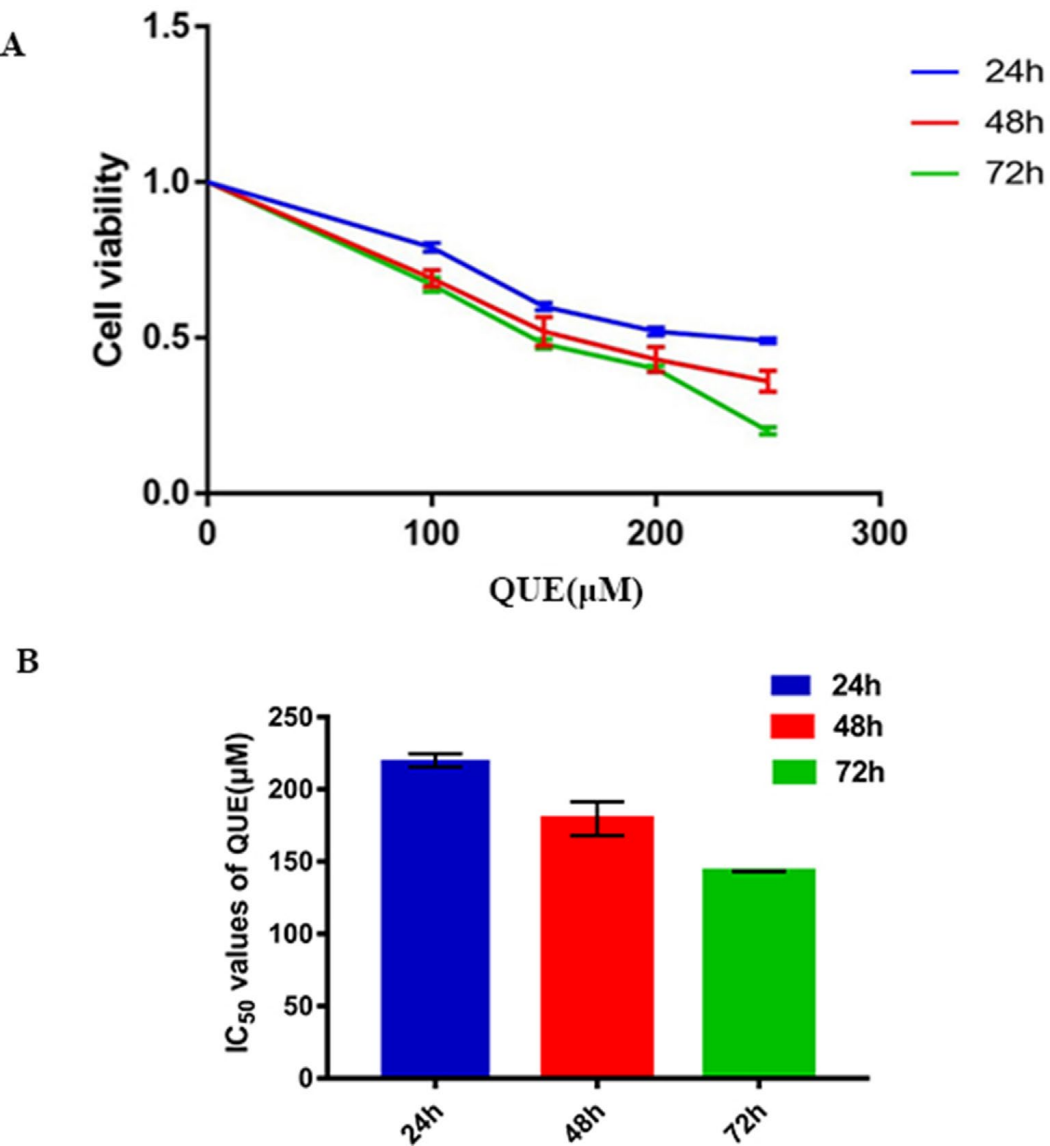
Effect of QUE on the proliferation of HCT116 cells by MTS assay. HCT116 cells were treated with quercetin at the indicated concentrations for 24, 48, and 72 h. QUE suppresses viability of HCT116 cells **(A)**. IC_50_ values for QUE at 24, 48, and 72 h in HCT116 cells **(B)**. Data are presented as the mean ± SD from at least three independent experiments. **Abbreviation:** QUE, quercetin.

As shown in **Figure 1A**, QUE at concentrations of 100, 150, 200, and 250 μΜ was used to treat HCT-116 cells for 24, 48, and 72 h, respectively, and the results showed that the viability of HCT-116 cells was markedly reduced by QUE, in both a dose- and time-dependent manner. As shown in **Figure 1B**, the IC_50_ values for QUE were 220.1, 179.7, and 143.23 μΜ.

### QUE Induces Apoptosis in HCT-116 Cells

**Figure 2:**
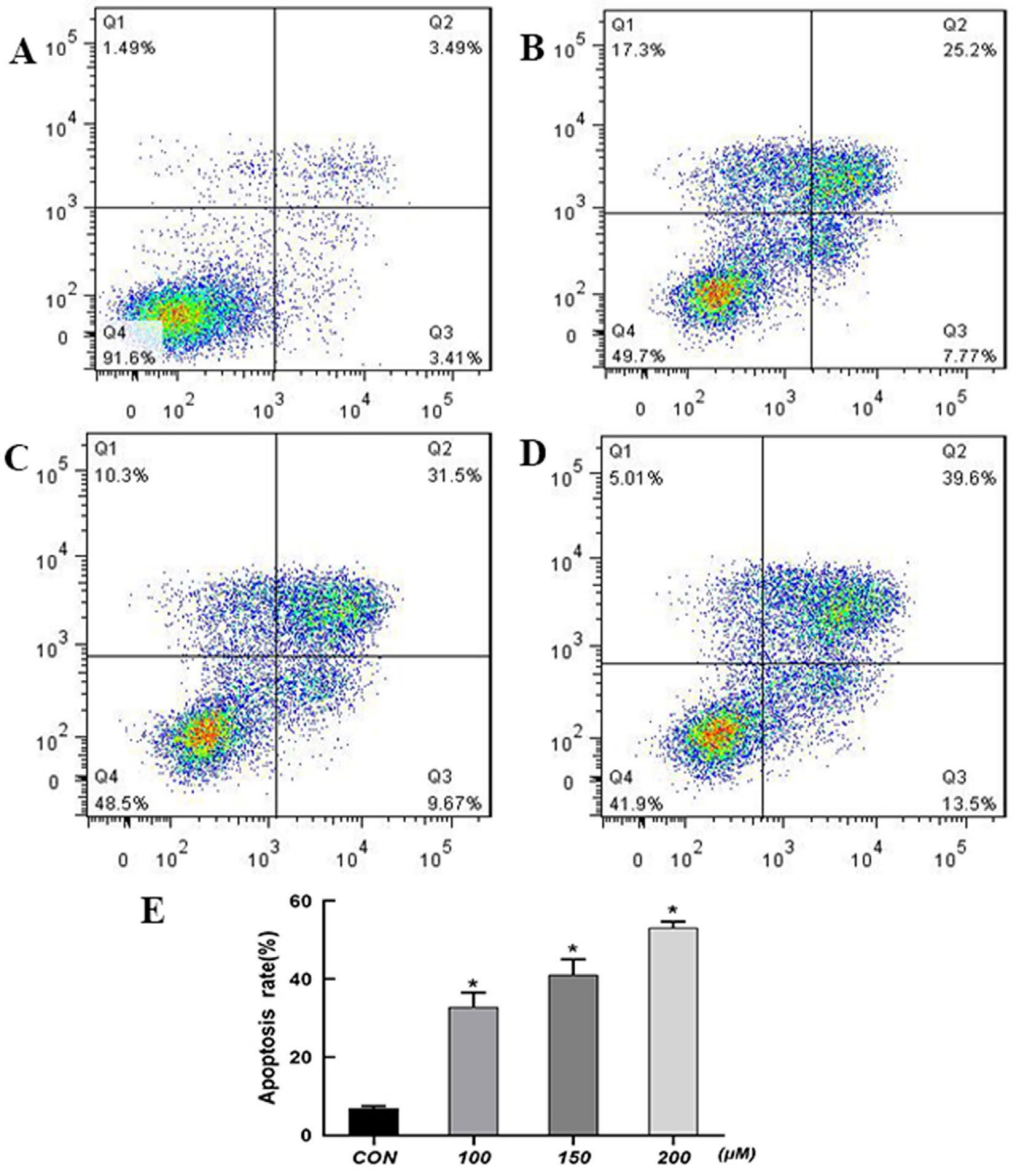
Effect of QUE at different concentrations on apoptosis of HCT116 cells as detected by flow cytometry. **(A)** Control; **(B)** 100 µM; **(C)** 150 µM; **(D)** 200 µM. **(E)** The percentage of apoptotic cells was presented as the mean ± SD of three independent experiments, *P < 0.05 vs. control; one-way ANOVA, followed by Dunnett’s *post hoc* test. **Abbreviations:** QUE, quercetin; CON, control.

Cells were treated with 100, 150, and 200 μM QUE for 72 h and stained with Annexin V-FITC and PI for the analysis of apoptosis. As shown in **Figure 2**, the percentage of apoptotic cells was significantly increased in a dose-dependent manner after QUE treatment. Taken together, these results clearly demonstrated QUE-induced apoptosis of HCT-116 cells.

### Sequencing and Mapping of the Transcriptome

The cDNA and sRNA libraries of cell samples from three groups of untreated HCT-116 cells and three groups of QUE-treated HCT-116 cells were sequenced. Moreover, counts of clean reads and mapped ratio of sequencing data are shown in **Table 2**.

**Table 2:** Deep RNA sequencing of subjects.

Sample ID		LncRNA + mRNA + cirRNA sequencing			miRNA sequencing		
		Clean reads	Q30 (%)	Mapped Ratio	Clean reads	Q30%	Mapped Ratio
QUE	QUE-1	136940730	93.8	76.42%	29589490	99.05	54.90%
	QUE-2	142365786	94.88	65.82%	33463913	99.11	60.02%
	QUE-3	116119138	94.98	58.93%	28926985	99.22	54.72%
CON	CON-1	117429248	94.4	78.85%	39189854	99.16	63.70%
	CON-2	130625532	95.01	78.88%	51489576	99.10	64.54%
	CON-3	118788322	94.63	77.92%	52661569	99.11	68.34%

QUE-1, QUE-2, and QUE-3 represent three groups of HCT-116 cells treated with QUE. CON-1, CON-2, and CON-3 represent three groups of untreated HCT-116 cells.

QUE, quercetin; CON, control.

### Differentially Expressed ncRNAs and mRNAs

**Figure 3:**
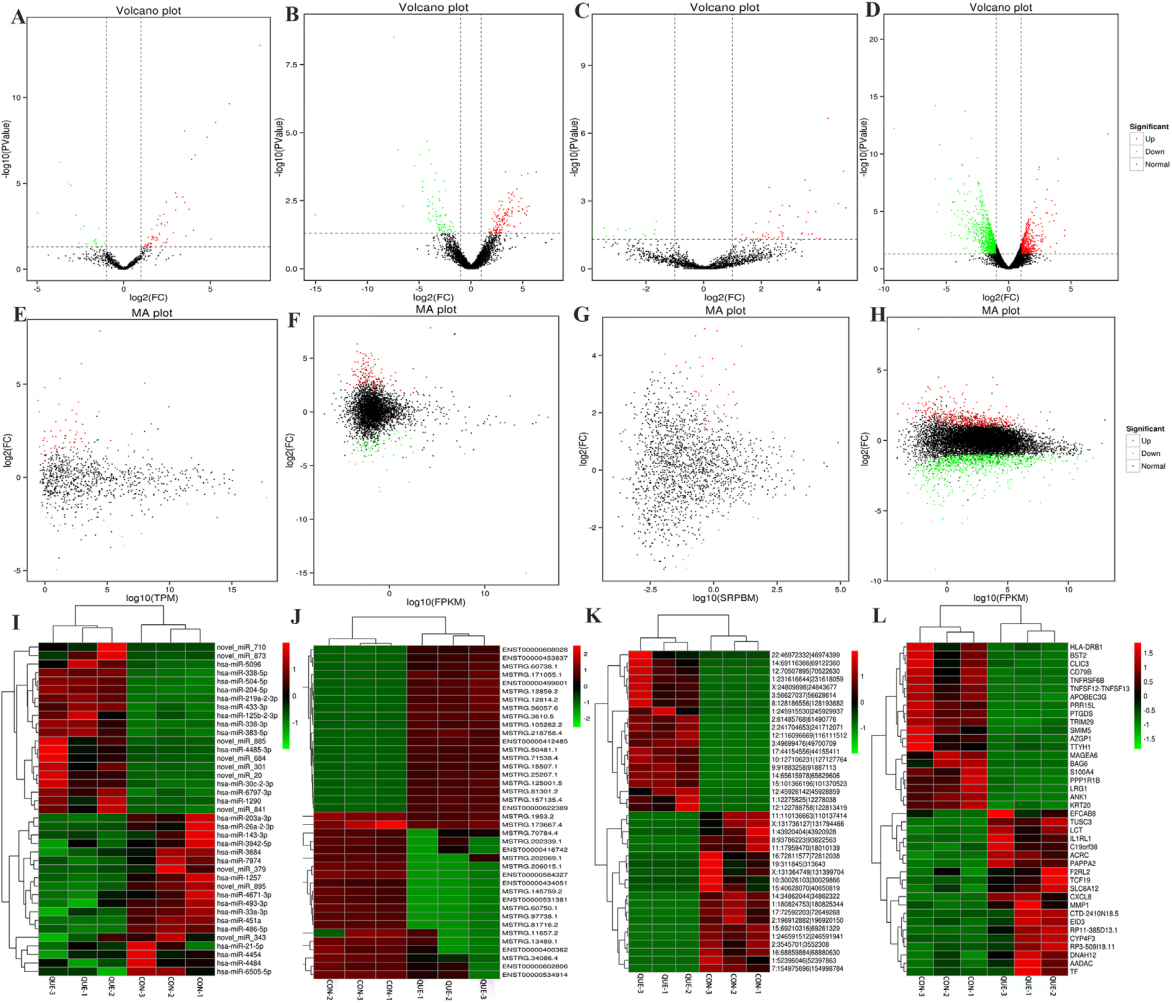
RNA-seq reveals distinct expression pattern of miRNAs, lncRNAs, circRNAs, and mRNAs in HCT116 cells and QUE-treated HCT116 cells. **(A–D)** Volcano plot of DEmiRNAs, DElncRNAs, DEcircRNAs, and DEmRNAs expression profiles between HCT116 cells and QUE-treated HCT116 cells. **(E-H)** MA plot of DEmiRNAs, DElncRNAs, DEcircRNAs, and DEmRNAs expression profiles between HCT116 cells and QUE-treated HCT116 cells. **(I–L)** Unsupervised clustering analysis showing expression profiles of top 40 differentially expressed miRNAs, lncRNAs, circRNAs, and mRNAs between HCT116 cells and QUE-treated HCT116 cells. QUE-1, QUE-2, and QUE-3 represent three groups of HCT-116 cells treated with QUE. CON-1, CON-2, and CON-3 represent three groups of untreated HCT-116 cells. **Abbreviation:** QUE, quercetin. CON, control.

Information of the top 20 up-regulated and 20 down-regulated lncRNAs, circRNAs, miRNAs, and mRNAs in the QUE-treated cells compared with the untreated cells are listed in **Tables 3**–**6**. Volcano plot, MA plot, and Unsupervised clustering analysis were applied to exhibit DEncRNA and DEmRNA expression profiles between the QUE-treated cells and the untreated cells. **Figures 3A–D** shows in detail the volcano plot of DEmiRNA, DElncRNA, DEcircRNA, and DEmRNA expression profiles, respectively. **Figures 3E–H** indicates the MA plot of DEmiRNA, DElncRNA, DEcircRNA, and DEmRNA expression profiles, respectively. The Unsupervised clustering analysis showing the expression profiles of the top 20 up- and down-regulated DEmiRNAs, DElncRNAs, DEcircRNAs, and DEmRNAs is illustrated in **Figures 3I–L**.

**Table 3:** The top 20 up- and down-regulated miRNAs.

Name	QUE-1	QUE-2	QUE-3	CON-1	CON-2	CON-3	*P* value	log2FC	Regulated
hsa-miR-219a-2-3p	47.8313	38.034	41.278	0	0.3117	0.0359	9.24E−14	7.8505	Up
novel_miR_20	10.7557	10.9283	23.3697	0.1065	0.1417	0.1435	2.33E−10	6.0999	Up
hsa-miR-338-3p	5.188	3.1838	5.3344	0	0	0.1793	2.87E−09	5.3058	Up
novel_miR_710	110	1155.4	265.3222	5.1665	12.5832	8.141	0.01733	5.0431	Up
hsa-miR-383-5p	2.2777	1.4628	2.0321	0.1598	0	0	2.09E−08	4.8215	Up
hsa-miR-30c-2-3p	1.0123	1.0326	1.9051	0	0.1417	0	2.30E−07	4.1288	Up
novel_miR_873	12.4007	22.8032	2.0321	0.1065	0.4534	0.7173	0.005265	4.0298	Up
hsa-miR-433-3p	4.6819	4.3885	3.4292	0.5326	0	0.0717	3.95E−07	3.9197	Up
novel_miR_885	790.6	1696.2	3132.3	108.4427	88.5639	54.7634	0.000332	3.7825	Up
hsa-miR-4485-3p	3.5431	5.9374	16.3842	0.4261	0.6235	0.251	0.011976	3.6025	Up
hsa-miR-1290	12.2742	57.8255	45.0883	2.024	2.2672	1.6139	0.000134	3.5209	Up
hsa-miR-6797-3p	1.0123	1.5489	1.5241	0.2131	0	0.0359	8.96E−09	3.5168	Up
hsa-miR-504-5p	11.2619	9.8957	11.4308	0.9055	0.9069	0.251	6.33E−05	3.4046	Up
novel_miR_301	3.29	6.0235	8.3826	0.6392	0.3968	0.2869	6.23E−05	3.0738	Up
hsa-miR-338-5p	9.6169	10.6702	10.7958	0.9587	1.3603	0.251	0.000364	2.9959	Up
novel_miR_841	2.0246	4.6467	3.4292	0.3728	0.4251	0.0359	3.64E−05	2.9880	Up
novel_miR_684	334.9454	508.6404	1209.8	60.1335	69.8875	41.6374	0.007656	2.8662	Up
hsa-miR-204-5p	7.3392	6.6258	8.1286	0.6924	0.9352	0.7531	0.001859	2.5244	Up
hsa-miR-125b-2-3p	6.0738	2.4094	4.1913	0.9055	0.2834	0.3586	0.017467	2.5090	Up
hsa-miR-5096	3.6696	2.4954	1.3971	0.2663	0.4534	0.1793	0.025683	2.4837	Up
hsa-miR-1257	0	0.2581	0.381	3.1958	2.2672	5.3078	0.000527	−4.9679	Down
hsa-miR-143-3p	167.4094	148.608	158.3804	222.4781	171.573	298.3478	0.046004	−1.2611	Down
hsa-miR-203a-3p	1965.3	2110.8	2034.6	2944.9	3156.6	3924.2	0.004354	−1.4364	Down
hsa-miR-21-5p	227730	184152	145194	259839	384154	139555	0.030963	−1.0935	Down
hsa-miR-26a-2-3p	1.0123	1.1186	0.8891	2.0772	2.9191	5.4154	0.030104	−2.5689	Down
hsa-miR-33a-3p	3.6696	3.0978	2.0321	5.6458	5.4697	6.4913	0.024884	−1.6717	Down
hsa-miR-3684	1.0123	1.2047	0.7621	2.1305	1.332	2.0801	0.030702	−1.5871	Down
hsa-miR-3942-5p	1.5185	0.6884	0.254	1.8109	1.502	3.2277	0.035983	−2.0560	Down
hsa-miR-4454	8.9842	9.3794	2.9212	9.4275	22.1055	11.8349	0.021729	−1.6904	Down
hsa-miR-4484	0	0.6884	1.2701	1.4381	3.2025	1.5421	0.0045	−2.4779	Down
hsa-miR-451a	18.2214	17.6402	18.4163	155.3671	117.6128	152.9216	5.91E−07	−3.6597	Down
hsa-miR-4671-3p	0.3796	0.9465	0.508	1.4381	1.0486	2.2235	0.033673	−2.1636	Down
hsa-miR-486-5p	16.5764	20.0496	31.2443	138.8024	117.3578	118.8514	1.12E−05	−3.1721	Down
hsa-miR-493-3p	0.8858	1.6349	1.1431	2.0772	2.0122	2.4746	0.03867	−1.5905	Down
hsa-miR-6505-5p	4.0492	1.9791	4.0643	7.6698	7.7653	4.8057	0.018848	−1.6118	Down
hsa-miR-7974	35.6836	83.2962	99.4482	491.8272	206.0634	370.2898	1.33E−05	−3.0231	Down
novel_miR_343	0.5062	1.1186	0.127	1.5979	1.0203	0.7531	0.029758	−1.5914	Down
novel_miR_379	11.3884	14.8006	11.9389	62.3173	20.6035	37.1904	0.00321	−2.3063	Down
novel_miR_895	1.645	2.4954	1.7781	7.8829	6.5466	9.5755	0.00074	−2.7583	Down

QUE-1, QUE-2, and QUE-3 represent three groups of HCT-116 cells treated with QUE. CON-1, CON-2, and CON-3 represent three groups of untreated HCT-116 cells.

QUE, quercetin; CON, control.

**Table 4:** The top 20 up- and down-regulated circRNAs.

NAME	QUE-1	QUE-2	QUE-3	CON-1	CON-2	CON-3	*P* value	log2FC	Regulated
2:61485768|61490776	2.8997	3.8419	3.5119	0	0	0	1.78E−11	Inf	Up
14:65615978|65629606	1.5727	1.328	1.6389	0	0	0	2.77E−06	Inf	Up
17:44154556|44155411	0.983	0.9012	1.2877	0	0	0	9.08E−05	Inf	Up
12:45926142|45928859	1.0321	1.1858	0.4683	0	0	0	0.000209	Inf	Up
10:127106231|127127764	0.9338	0.7589	1.1121	0	0	0	0.000246	Inf	Up
1:12275825|12278038	0.5406	1.328	0.8195	0	0	0	0.00025	Inf	Up
1:231616644|231618059	0.7372	0.7115	1.2292	0	0	0	0.000382	Inf	Up
14:69116366|69122360	0.7372	0.4743	1.3462	0	0	0	0.000599	Inf	Up
12:70507895|70522630	0.6881	0.5217	1.1706	0	0	0	0.000865	Inf	Up
9:91883258|91887113	0.7864	0.6166	0.7024	0	0	0	0.001363	Inf	Up
12:116096669|116111512	0.5898	0.8063	0.5853	0	0	0	0.001712	Inf	Up
2:241704653|241712071	0.5406	0.7115	0.7024	0	0	0	0.002002	Inf	Up
22:46972332|46974399	0.4915	0.332	2.2242	0	0	0	0.002672	Inf	Up
15:101366196|101370523	0.6389	0.4743	0.6439	0	0	0	0.003471	Inf	Up
X:24809898|24843677	0.3932	0.4743	0.8195	0	0	0	0.004631	Inf	Up
12:122788758|122813419	0.344	0.8063	0.4097	0	0	0	0.004876	Inf	Up
3:49699476|49700709	0.5406	0.5692	0.4683	0	0	0	0.005033	Inf	Up
1:245915530|245929937	0.8355	0.2846	0.4683	0	0	0	0.005185	Inf	Up
3:56627037|56628614	0.3932	0.4269	0.8195	0	0	0	0.00532	Inf	Up
8:128186556|128193682	0.3932	0.4269	0.8195	0	0	0	0.00532	Inf	Up
14:34862044|34862322	0	0	0	1.4376	0.9824	1.0778	2.03E−05	#NAME?	Down
17:72592203|72649268	0	0	0	0.9776	0.7756	0.5673	0.000559	#NAME?	Down
10:30026103|30029866	0	0	0	0.805	2.3784	0.2836	0.000642	#NAME?	Down
19:311845|313643	0	0	0	0.23	1.4477	0.5673	0.001014	#NAME?	Down
8:93786223|93822563	0	0	0	0.575	0.6204	0.9076	0.001214	#NAME?	Down
2:3545701|3552308	0	0	0	0.46	0.7756	0.7942	0.001532	#NAME?	Down
15:69210316|69261329	0	0	0	0.575	0.6721	0.7374	0.001611	#NAME?	Down
1:180824753|180825344	0	0	0	0.7475	0.6204	0.5673	0.001657	#NAME?	Down
1:246591512|246591941	0	0	0	0.46	0.5687	0.5673	0.004485	#NAME?	Down
15:40628070|40650819	0	0	0	0.46	0.8273	0.1135	0.007056	#NAME?	Down
11:110136663|110137414	0	0	0	0.575	0.2585	0.5673	0.007095	#NAME?	Down
1:43920404|43920928	0	0	0	0.4025	0.2068	0.7374	0.008786	#NAME?	Down
16:72811577|72812038	0	0	0	0.23	0.7756	0.3404	0.008888	#NAME?	Down
1:52396046|52397863	0	0	0	0.4025	0.6204	0.2269	0.010575	#NAME?	Down
16:68859884|68880630	0	0	0	0.2875	0.517	0.4538	0.010939	#NAME?	Down
7:154975696|154998784	0	0	0	0.4025	0.4653	0.2836	0.01359	#NAME?	Down
11:17959470|18010139	0	0	0	0.2875	0.3102	0.5105	0.015873	#NAME?	Down
X:131736127|131794466	0	0	0	0.4025	0.1551	0.4538	0.019845	#NAME?	Down
X:131364749|131399704	0	0	0	0.115	0.5687	0.2836	0.021384	#NAME?	Down
2:196912882|196920150	0	0	0	0.4025	0.2585	0.2269	0.026239	#NAME?	Down

QUE-1, QUE-2, and QUE-3 represent three groups of HCT-116 cells treated with QUE. CON-1, CON-2, and CON-3 represent three groups of untreated HCT-116 cells.

QUE, quercetin; CON, control.

**Table 5:** The top 20 up- and down-regulated lncRNAs.

#ID	QUE-1	QUE-2	QUE-3	CON-1	CON-2	CON-3	*P* value	log2FC	Regulated
MSTRG.56057.6	1.011199	0.564535	0.780016	0	0	0	1.42E−06	Inf	Up
MSTRG.12859.2	0.491669	0.61381	0.90822	0	0	0	2.09E−06	Inf	Up
MSTRG.3610.5	0.86471	0.78501	0.372459	0	0	0	7.70E−06	Inf	Up
MSTRG.218758.4	0.687719	1.616394	0.17021	0	0	0	9.68E−06	Inf	Up
ENST00000499601	0.524056	0.821801	1.320079	0	0	0	1.24E−05	Inf	Up
ENST00000412485	0.541436	0.442815	0.133381	0	0	0	1.61E−05	Inf	Up
MSTRG.71538.4	0.362249	0.312796	0.115272	0	0	0	2.42E−05	Inf	Up
MSTRG.171055.1	1.421666	0.22262	0.939521	0	0	0	2.45E−05	Inf	Up
MSTRG.105262.2	0.845969	0.384586	0.337466	0	0	0	3.03E−05	Inf	Up
MSTRG.81301.2	0.314015	0.455942	0.181373	0	0	0	3.19E−05	Inf	Up
MSTRG.157135.4	0.370713	0.661509	0.264077	0	0	0	4.17E−05	Inf	Up
ENST00000622389	0.417104	0.54098	0.30028	0	0	0	8.36E−05	Inf	Up
MSTRG.15507.1	0.29734	0.287354	0.132048	0	0	0	0.000133	Inf	Up
ENST00000453837	0.076754	0.195031	0.132529	0	0	0	0.000176	Inf	Up
ENST00000608028	0.133745	0.067203	0.059525	0	0	0	0.000225	Inf	Up
MSTRG.125001.5	0.334434	0.361555	0.408215	0	0	0	0.000265	Inf	Up
MSTRG.25207.1	0.170071	0.553006	0.210474	0	0	0	0.000266	Inf	Up
MSTRG.12814.2	0.381843	0.453598	0.957113	0	0	0	0.000396	Inf	Up
MSTRG.60738.1	0.090811	0.30324	0.296676	0	0	0	0.000415	Inf	Up
MSTRG.50481.1	0.481522	0.216593	0.167346	0	0	0	0.000578	Inf	Up
MSTRG.206015.1	0	0	0	0.8716	0.9477	1.1035	1.61E−06	#NAME?	Down
MSTRG.97738.1	0	0	0	0.1847	0.4755	0.3154	0.000217	#NAME?	Down
MSTRG.81716.2	0	0	0	0.2771	0.2757	0.4464	0.000119	#NAME?	Down
MSTRG.145759.2	0	0	0	0.4361	0.3396	0.135	2.09E−06	#NAME?	Down
MSTRG.60750.1	0	0	0	0.5182	0.6108	0.3925	1.34E−09	#NAME?	Down
ENST00000584327	0	0	0	3.0625	1.5157	4.1065	1.19E−09	#NAME?	Down
ENST00000531381	0	0	0	0.1968	0.1891	0.1018	1.19E−06	#NAME?	Down
ENST00000434051	0	0	0	0.1172	0.0638	0.0642	2.69E−06	#NAME?	Down
MSTRG.173667.4	1.7892	1.5229	2.3347	1.5777	354.65	123556	0.010557	−15.0164	Down
MSTRG.34086.4	0.0033	0.0004	0	1.2649	1.1295	0.714	3.13E−09	−7.4146	Down
ENST00000400362	0.0271	0	0	1.9207	0.2462	0.727	4.26E−05	−7.0340	Down
MSTRG.11657.2	0.5142	0	0	0	9.3146	15.1451	0.005018	−6.5397	Down
ENST00000534914	0.0495	0.1393	0	0.6644	2.3301	1.8312	0.000169	−5.4299	Down
MSTRG.70784.4	0	0.0129	0.005	0.1356	0.1773	0.1709	6.48E−05	−4.8623	Down
ENST00000602806	0.0228	0.0614	0	0.6543	0.3518	0.6894	0.00142	−4.6659	Down
ENST00000416742	0	0.0165	0	0.1446	0.0889	0.0773	0.001011	−4.6632	Down
MSTRG.202069.1	0	0	0.0392	0.1394	0.3821	0.3348	0.000617	−4.6500	Down
MSTRG.1953.2	0.4877	0.4417	0.422	7.8476	4.2212	6.9376	2.10E−05	−4.2371	Down
MSTRG.202339.1	0	0.0958	0	0.2168	0.5654	0.2458	0.00507	−4.1651	Down
MSTRG.13489.1	0.3488	0	0	0.8859	1.1009	1.6577	0.002828	−4.1369	Down

QUE-1, QUE-2, and QUE-3 represent three groups of HCT-116 cells treated with QUE. CON-1, CON-2, and CON-3 represent three groups of untreated HCT-116 cells.

QUE, quercetin; CON, control.

**Table 6:** The top 20 up- and down-regulated mRNAs.

Gene Symbol	QUE-1	QUE-2	QUE-3	CON-3	CON-2	CON-1	*P* value	log2FC	Regulated
RP11-385D13.1	0.459	0.5438	0.1785	0.003	0	0	1.82E−12	7.9515	Up
CTD-2410N18.5	40.0383	37.9792	2.5737	0.15	0.2501	1.9876	0.001324	4.4775	Up
CXCL8	41.3213	20.8464	35.8399	1.6461	1.6596	2.5119	2.21E−08	3.9679	Up
IL1RL1	0.4132	0.5353	0.9505	0.0603	0.0438	0.0062	1.53E−05	3.8184	Up
EFCAB8	0.2146	0.4772	1.0995	0.0334	0.2802	0.0354	0.032473	3.7396	Up
ACRC	2.2348	1.6744	2.5938	0.3101	0.0706	0.273	1.27E−07	3.0842	Up
PAPPA2	0.3623	0.3721	0.5551	0.0637	0.0413	0.0333	1.39E−05	3.0253	Up
TUSC3	2.2369	3.4756	2.6733	0.1932	0.236	0.1381	8.89E−07	2.9856	Up
EID3	7.5414	6.3091	1.5003	1.0693	0.3949	0.263	0.001709	2.9377	Up
MMP1	4.5738	1.8258	1.8633	0.1547	0.5818	0.223	0.001026	2.7474	Up
AADAC	7.5793	4.7235	1.7104	1.4535	0.2833	0.2375	0.004429	2.7197	Up
CYP4F3	12.0443	13.418	2.9752	1.8013	1.9014	0.9014	0.002971	2.7178	Up
TCF19	0.6208	1.3387	0.4906	0.1369	0.1059	0.068	0.000219	2.6952	Up
SLC6A12	0.3203	0.492	0.2143	0.1176	0.0204	0	0.004181	2.6693	Up
C19orf38	2.3469	2.4262	4.4076	0.0535	0.3851	0.6403	0.001546	2.6459	Up
DNAH12	0.1975	0.1442	0.0795	0.047	0.0126	0.0039	0.001165	2.6171	Up
F2RL2	0.2827	0.6668	0.2137	0.2595	0.0422	0	0.004704	2.6124	Up
LCT	0.3575	0.5997	0.6578	0.0971	0.049	0.0792	0.000116	2.6094	Up
TF	2.1888	1.3499	0.1846	0.2799	0.061	0.1333	0.023651	2.5816	Up
RP3-509I19.11	1.0911	1.3781	0.2671	0.1918	0.0291	0.1481	0.002571	2.5804	Up
MAGEA6	0	0	0	1.724	0.7861	1.3604	6.11E−16	#NAME?	Down
TNFSF12-TNFSF13	0	0.0017	0	0.6983	1.3414	1.0317	6.26E−13	-9.1872	Down
ANK1	0	0.0032	0.0038	0.1533	0.1184	0.1713	1.51E−08	-5.8831	Down
PRR15L	0.1197	0.1226	0.0523	4.2384	6.2581	3.616	6.27E−15	-5.8829	Down
BAG6	0.0169	0.0162	0.0173	1.064	0.2955	1.0986	1.23E−05	-5.7552	Down
CD79B	0.0782	0.0434	0	0.7712	1.9847	1.7625	2.56E−06	-5.5688	Down
KRT20	0.1982	0.0153	0	1.2966	1.2261	1.7863	1.50E−08	-4.6740	Down
HLA-DRB1	0.766	0	0	1.7502	7.4034	4.9431	0.000763	-4.6398	Down
AZGP1	0.0997	0	0.0493	0.7883	2.0076	1.042	8.16E−06	-4.5796	Down
TTYH1	0.1016	0.0395	0	0.6519	1.6136	0.4997	0.001463	-4.5639	Down
APOBEC3G	0.0512	0	0.0447	0.4597	0.7297	0.6744	1.52E−06	-4.4927	Down
TNFRSF6B	21.537	22.9604	25.2291	236.6955	496.5118	462.5763	1.58E−06	-4.4295	Down
PPP1R1B	0.0538	0.0211	0.144	1.2598	1.2531	2.2257	1.90E−05	-4.3329	Down
S100A4	10.3514	11.3851	17.5961	152.9374	203.8127	277.9736	1.45E−06	-4.3277	Down
SMIM5	0.0333	0.1101	0.0115	0.8277	1.2896	0.4922	0.001183	-4.3099	Down
BST2	1.103	0.5154	1.395	7.6829	20.0847	14.3881	2.66E−05	-4.0807	Down
PTGDS	0.1114	0.1319	0.0823	1.2747	1.8475	1.4403	8.25E−06	-4.0613	Down
TRIM29	0.015	0.0954	0.0401	0.6682	0.8271	0.6418	1.71E−07	-4.0474	Down
LRG1	0.1242	0.1033	0.2308	1.7433	1.6964	2.5503	2.71E−07	-3.9326	Down
CLIC3	0.5461	0.6195	2.4456	7.4547	23.1513	16.0411	0.000133	-3.9134	Down

QUE-1, QUE-2, and QUE-3 represent three groups of HCT-116 cells treated with QUE. CON-1, CON-2, and CON-3 represent three groups of untreated HCT-116 cells.

QUE, quercetin; CON, control.

**Figure 4:**
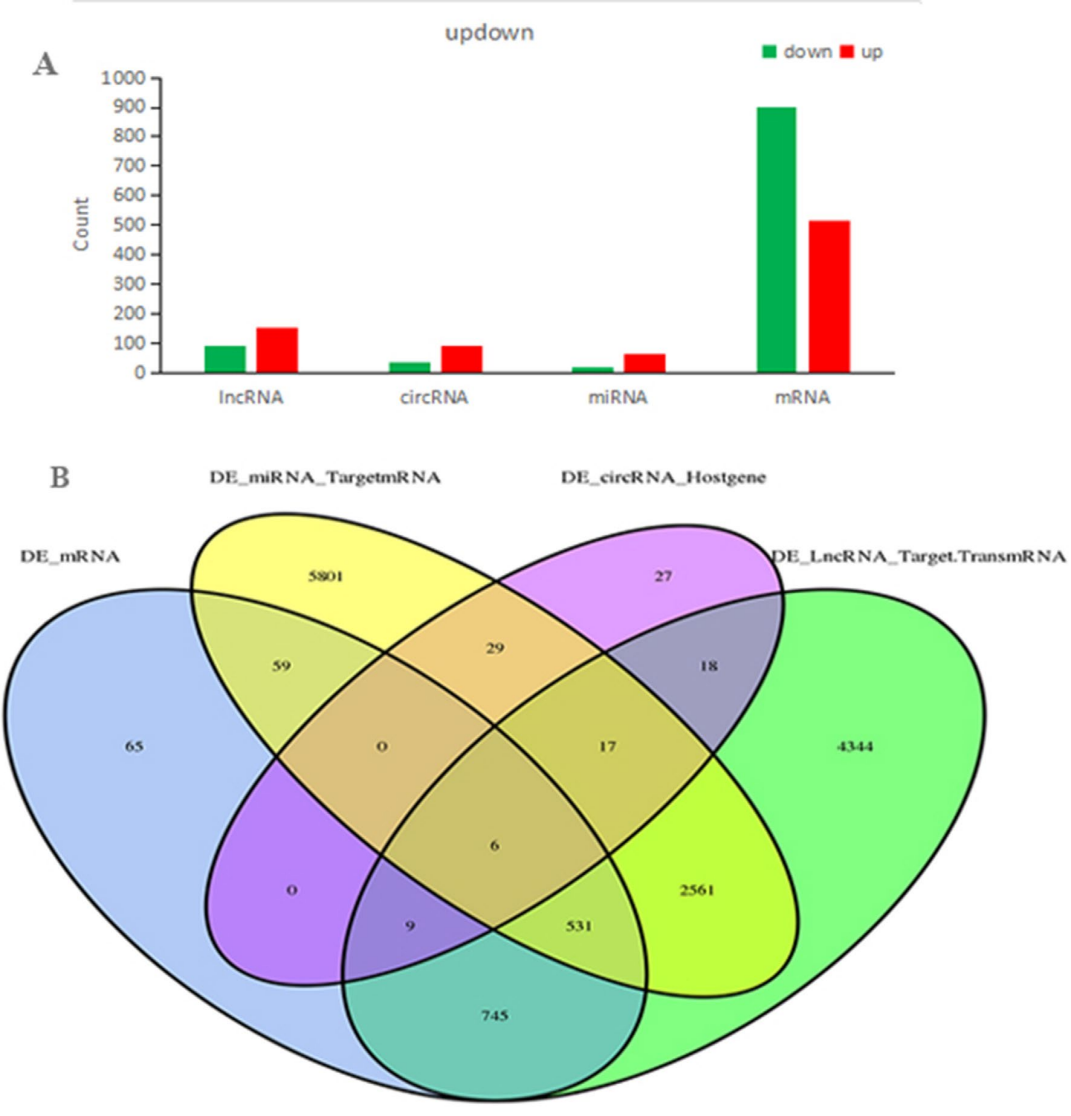
Count of relative differential expressed ncRNAs and mRNAs in HCT116 cells and QUE-treated HCT116 cells. Histogram showing the number of up- and down-regulated ncRNAs and mRNAs. Green represents up-regulation, and red represents down-regulation **(A)**. Venn diagram showing the overlap number of DE-mRNA, DE-LncRNA-Target.TransmRNA, DE-miRNA-TargetmRNA, and DE-circRNA-Hostgene **(B)**. **Abbreviation:** QUE, quercetin.

In total, our project detected 15,755 lncRNAs, 25,843 mRNAs, 2,735 miRNAs, and 6,911 new circRNAs. As shown in **Figure 4A**, there were 240 DElncRNAs (89 down-regulation and 151 up-regulation), 131 DEcircRNAs (37 down-regulation and 94 up-regulation), 83 DEmiRNAs (19 down-regulation and 64 up-regulation), and 1,415 DEmRNAs (901 down-regulation and 514 up-regulation) in the QUE-treated cells compared to the untreated cells, respectively. In an effort to further pinpoint the genes involved in the mechanism of QUE anti-CRC, we performed a global overlapping gene analysis. The intersections of DEmRNA, DEmiRNA–Target mRNA, DELncRNA–Target Trans.mRNA, and DEcircRNA–Hostgene are showed in **Figure 4B**. This left us with six reliable core mRNAs validated by different platforms and across different institutions, including ENSG00000101187, ENSG00000111344, ENSG00000116183, ENSG00000125319, ENSG00000150637, and ENSG00000169035.

### Expression Profile Validation

**Figure 5:**
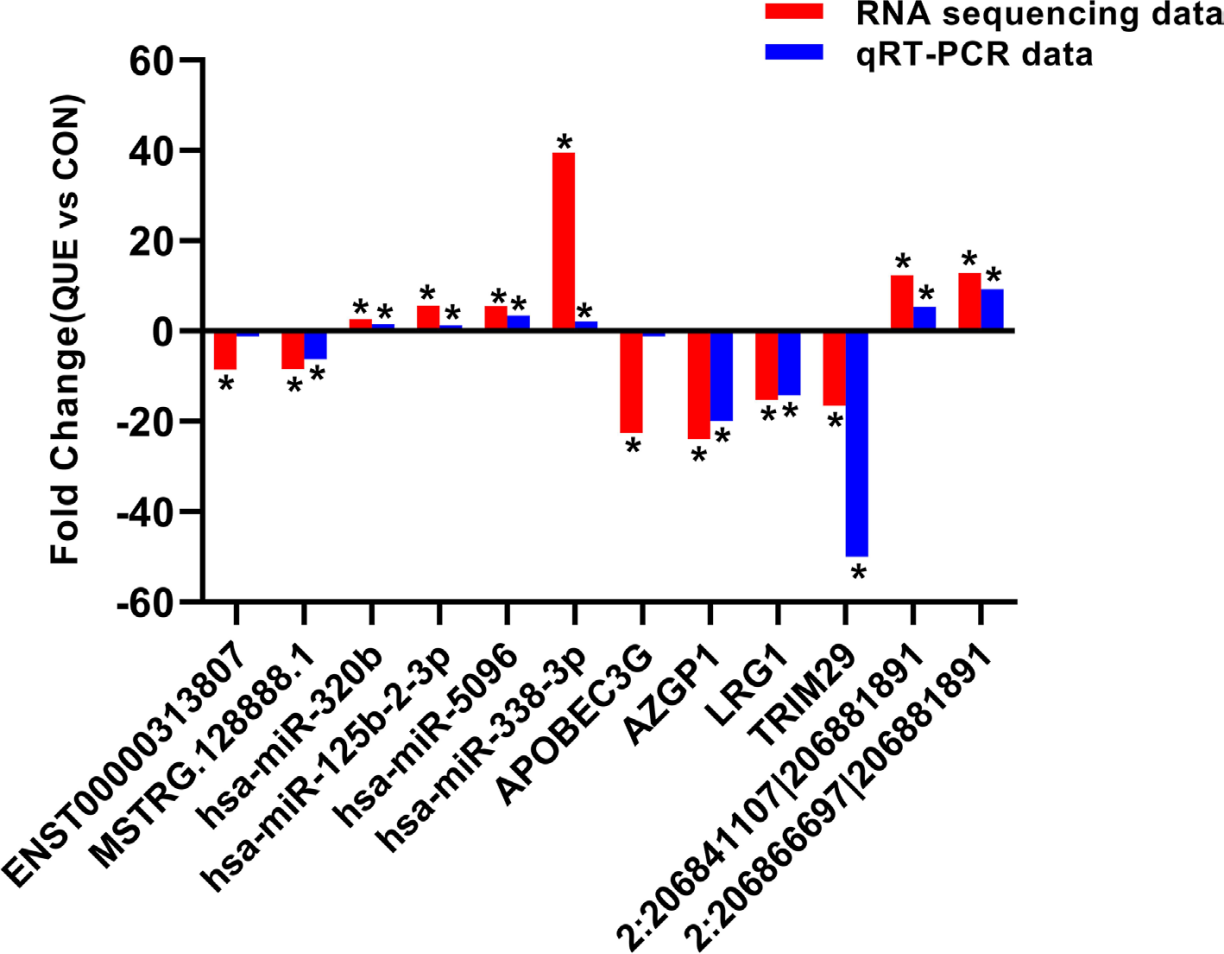
The differential expression of ncRNAs and mRNAs was validated by quantitative real-time PCR (qRT-PCR). The data showed that the expression levels of the hsa-miR-125b-2-3p, hsa-miR-338-3p, hsa-miR-320b, hsa-miR-5096, circRNA 2:206841107|206881891, and circRNA 2:206866697|206881891 were up-regulated and that the LRG1, AZGP1, TRIM29, APOBEC3, lncRNA ENST00000313807, and lncRNA MSTRG.128888.1 were down-regulated in QUE-treated HCT116 cells relative to the HCT116 cells. The heights of the columns in the chart represent fold change. The qRT-PCR results were consistent with the RNA sequencing data. *P < 0.05, QUE vs. CON. QUE represents HCT-116 cells treated with QUE. CON represents untreated HCT-116 cells. **Abbreviations:** QUE, quercetin. CON, control.

To validate the accuracy and reliability of the RNA sequencing results, a total of 12 dysregulated ncRNAs and mRNAs were selected for qRT-PCR analysis, including four miRNAs (hsa-miR-125b-2-3p, hsa-miR-338-3p, hsa-miR-320b, and hsa-miR-5096), four mRNAs (LRG1, AZGP1, TRIM29, and APOBEC3), two lncRNAs (ENST00000313807 and MSTRG.128888.1), and two circRNAs (2:206841107|206881891 and 2:206866697|206881891). As shown in **Figure 5**, the results from sequencing data were in agreement with those from qRT-PCR in terms of the expression levels of the validated ncRNAs and mRNAs.

### GO Enrichment Analysis

**Figure 6:**
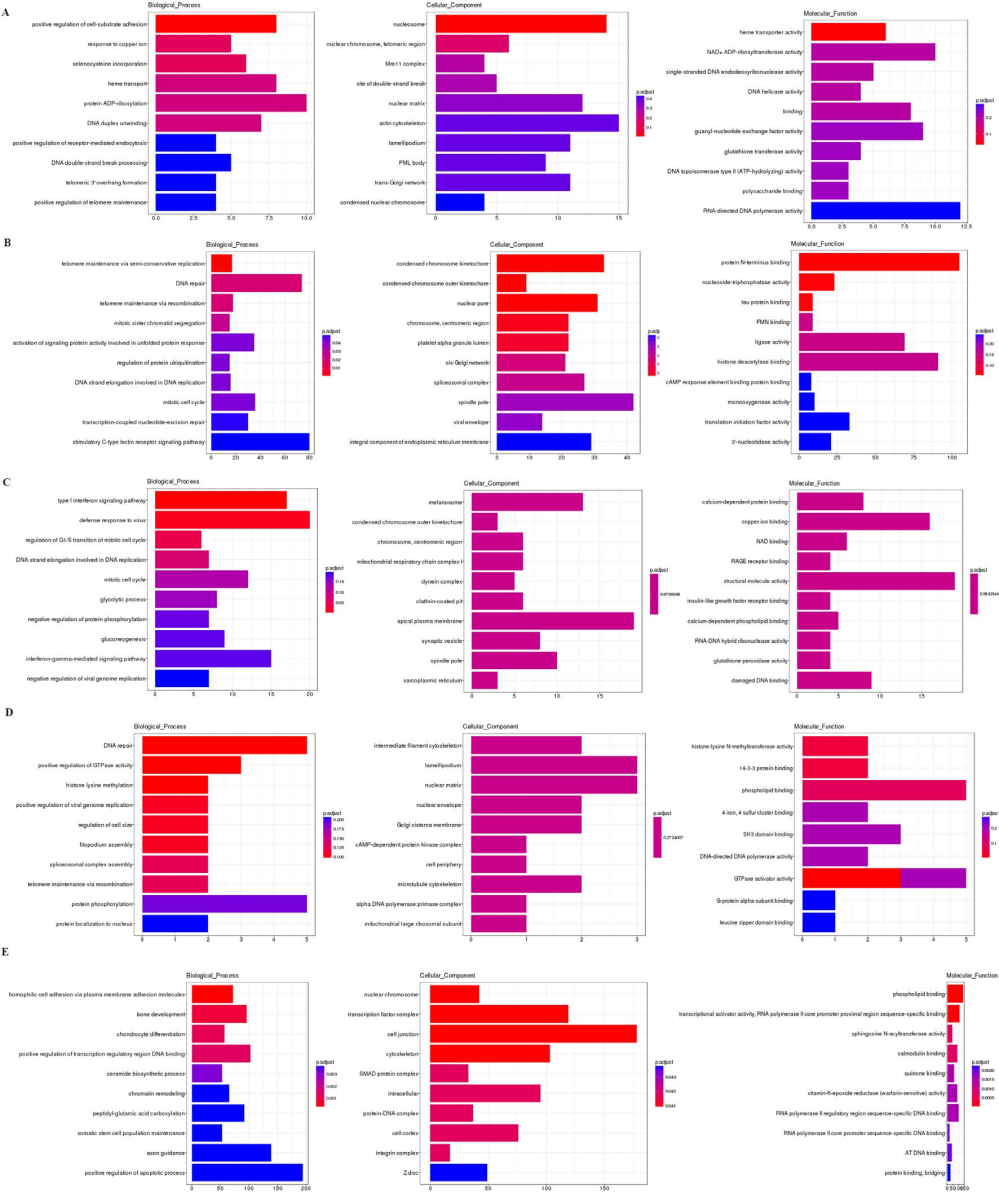
The GO enrichment analysis of the cis targeted genes of DE lncRNA **(A)**, the trans targeted genes of DE lncRNA **(B)**, DE mRNA **(C)**, hostgenes of DE cirRNA **(D)**, and targeted genes of DE miRNA **(E)**. The GO enrichment analysis provided a controlled vocabulary to describe the co-expressed genes of the differentially expressed ncRNAs and mRNA. The ontology covered three domains: biological process, cellular component, and molecular function. The abscissa represents the number of genes annotated in the GO term, the ordinate represents the GO term, and the color of the column represents the corrected *p* value. **Abbreviation:** GO, Gene Ontology.

Based on the GO enrichment analysis of the cis targeted genes of lncRNA (**Figure 6A**), the most significantly enriched BP, CC, and MF were protein ADP-ribosylation, actin cytoskeleton, and RNA-directed DNA polymerase activity, respectively. Based on the GO enrichment analysis of the trans targeted genes of lncRNA (**Figure 6B**), the most significantly enriched BP, CC, and MF were stimulatory C-type lectin receptor signaling pathway, spindle pole, and protein N-terminus binding, respectively. Based on the GO enrichment analysis of DEmRNAs (**Figure 6C**), the most significantly enriched BP, CC, and MF were defense response to virus, apical plasma membrane, and structural molecule activity, respectively. Based on the GO enrichment analysis of source genes of DEcircRNAs (**Figure 6D**), the most significantly enriched BP, CC, and MF were protein phosphorylation, nuclear matrix, and phospholipid binding, respectively. Based on the GO enrichment analysis of targeted genes of DEmiRNAs (**Figure 6E**), the most significantly enriched BP, CC, and MF were positive regulation of apoptotic process, cell junction, and phospholipid binding, respectively.

### KEGG Pathway Enrichment Analysis

**Figure 7:**
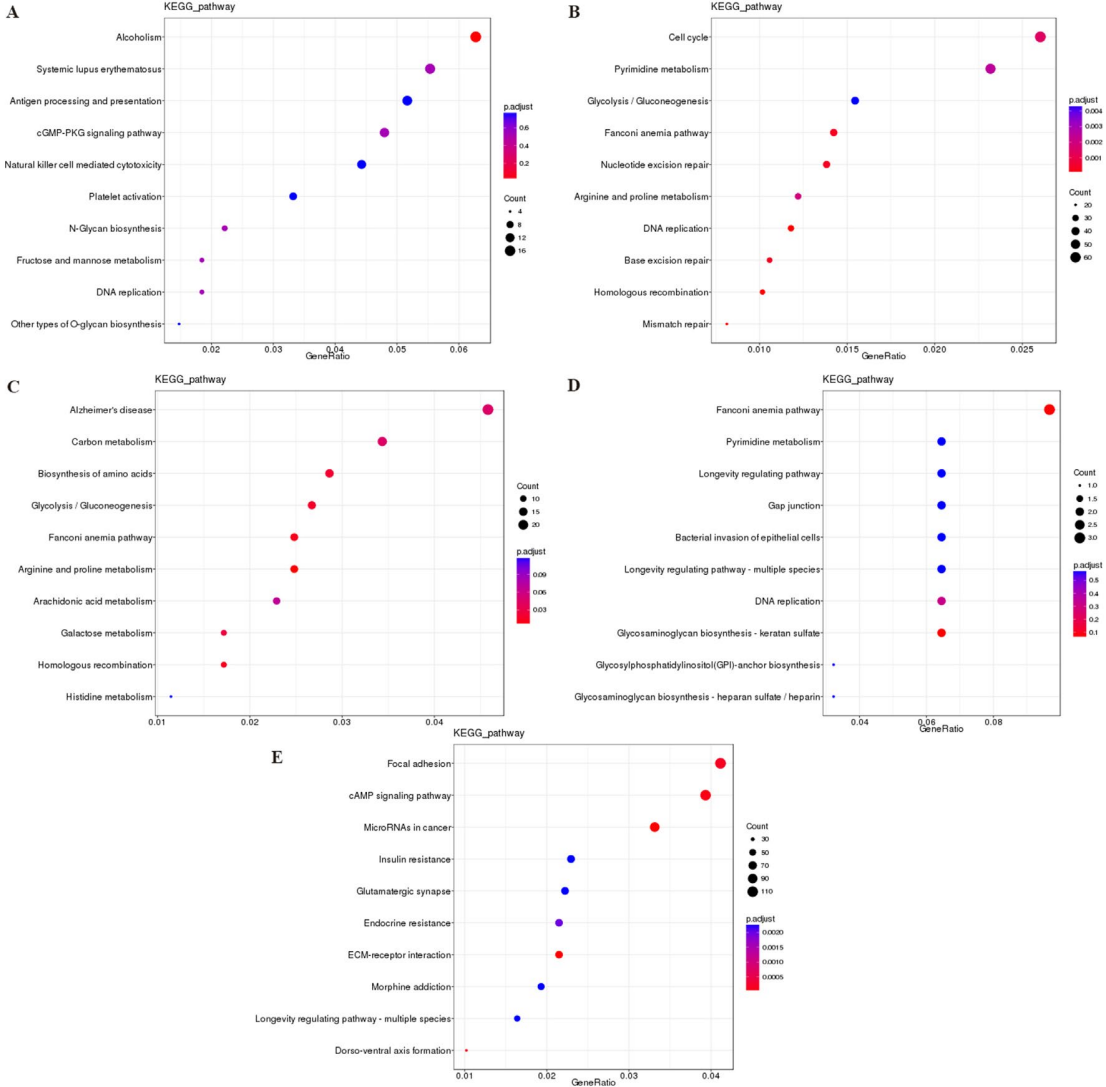
The KEGG enrichment analysis of the cis targeted genes of DElncRNA **(A)**, the trans targeted genes of DElncRNA **(B)**, DEmRNA **(C)**, hostgenes of DEcirRNA **(D)**, and targeted genes of DEmiRNA **(E)**. The abscissa GeneRatio represents the proportion of genes of interest in the pathway, and the ordinate represents each pathway. The size of the dots represents the number of genes annotated in the pathway, and the color of the dots represents the corrected *p* value of the hypergeometric test. **Abbreviation:** KEGG, Kyoto Encyclopedia of Genes and Genomes.

The most significantly enriched KEGG pathways are shown in **Figure 7**. For the cis targeted genes of DElncRNA (**Figure 7A**), alcoholism, systemic lupus erythematosus, and antigen processing and presentation were the most significant pathways for enrichment. For the trans targeted genes of DElncRNA (**Figure 7B**), cell cycle, pyrimidine metabolism, and glycolysis/gluconeogenesis were the most significant enriched pathway. For the DEmRNAs (**Figure 7C**), Alzheimer’s disease, carbon metabolism, and biosynthesis of amino acids were the most significant enriched pathway. For the hostgenes of DEcircRNA (**Figure 7D**), Fanconi anemia pathway, glycosaminoglycan biosynthesis–keratan sulfate, and DNA replication were the most significant enriched pathway. For the DE miRNAs (**Figure 7E**), focal adhesion, cAMP signaling pathway, and miRNAs in cancer were the most significant enriched pathway. The main biochemical pathways and signal transduction pathways determined by KEGG analysis will provide further insight into future research directions of ncRNAs and mRNA.

### Regulatory Network of ncRNAs and mRNA

**Figure 8:**
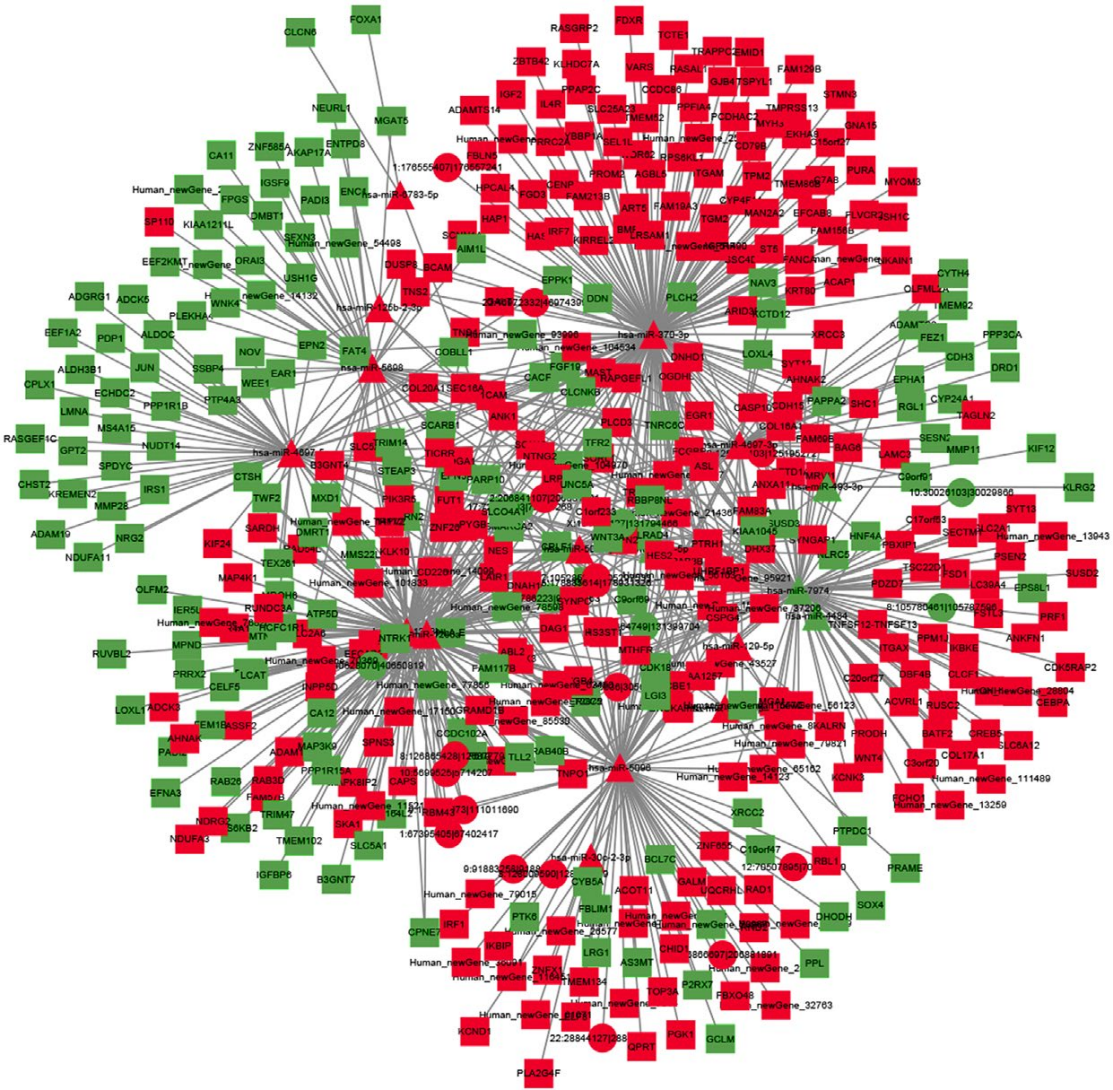
The interaction network of circRNA–miRNA–mRNA. Red and green represent up- and down-regulation, respectively.

**Figure 9:**
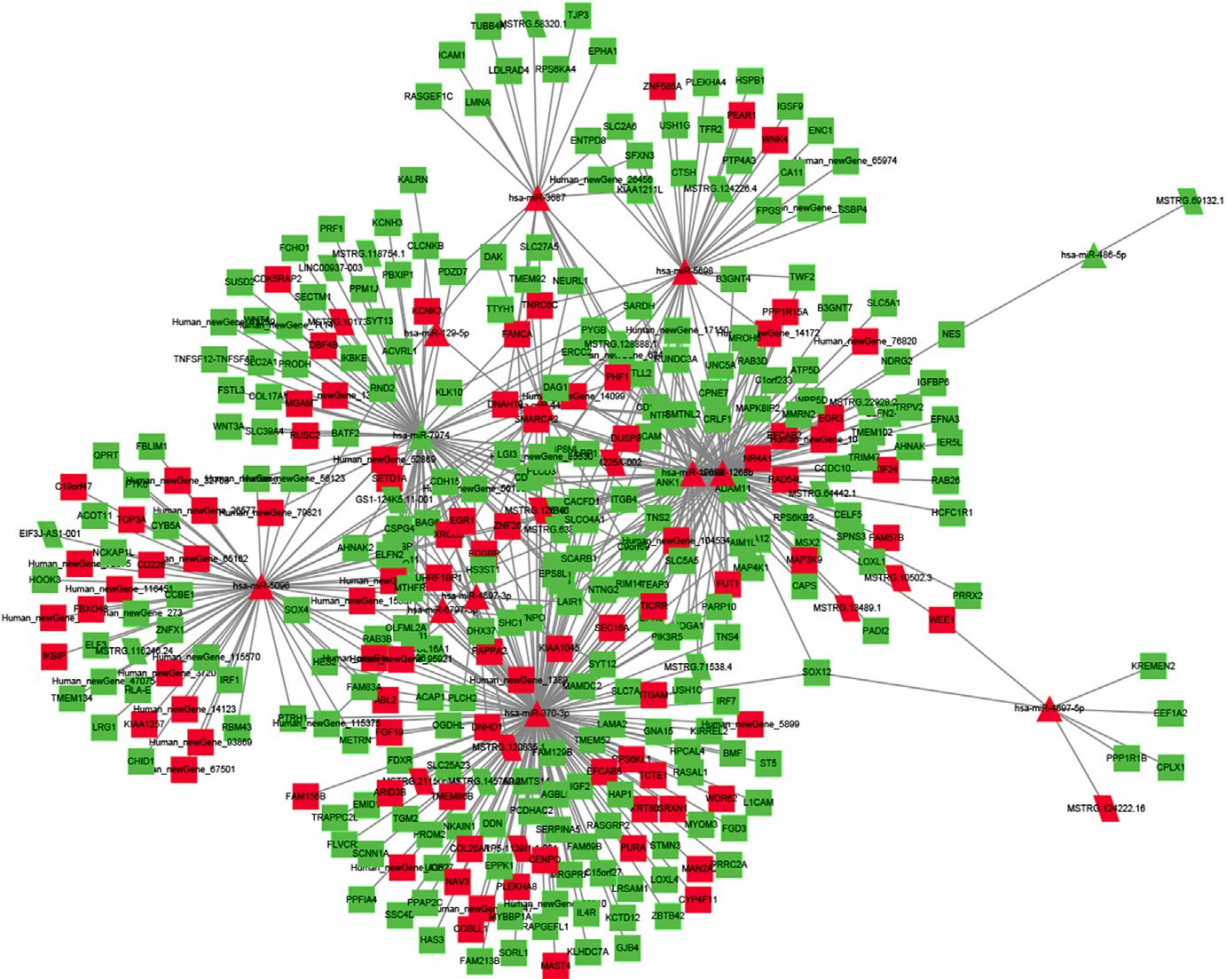
The interaction network of lncRNA–miRNA–mRNA. Red and green represent up- and down-regulation, respectively.

To explore the molecular mechanism of ncRNAs, a circRNA–miRNA–mRNA regulatory network and lncRNA–miRNA–mRNA network were constructed based on the theory of ceRNA. By using circRNA as a decoy, miRNA as the center, and mRNA as the target, the circRNA–miRNA–mRNA regulation network containing 437 mRNAs, 23 circRNAs, and 17 miRNAs was generated (**Figure 8**). By using lncRNA as a decoy, miRNA as center, and mRNA as target, the lncRNA–miRNA–mRNA regulation network that contained 331 mRNAs, 24 lncRNAs, and 13 miRNAs was built (**Figure 9**). In these two networks, different shapes represent different RNA types; red and green represent up- and down-regulation, respectively. Interestingly, circRNA (8:93786223|93822563), ENST00000313807, and ENST00000449307 were all able to regulate LRG1 expression, through competitively binding with miR-5096. These results suggested that circRNAs and lncRNAs harbor miRNA response elements and play pivotal regulatory roles in the mechanisms of QUE in anti-CRC.

## Discussion

CRC is one of the most common types of malignant tumors, ranking as the second leading cause of cancer-induced death worldwide. Multiple lines of studies have demonstrated the efficacy of QUE in CRC (Dihal et al., 2008; Refolo et al., 2015; Darband et al., 2018), but the exact mechanism of its anti-tumor effects in CRC remains unclear. To the best of our knowledge, this is the first comprehensive report of lncRNA, mRNA, circRNA, and miRNA to reveal regulator pathways with regard to QUE-induced apoptosis in HCT-116 cells.

In general, our data confirmed that QUE could inhibit the proliferation and induce apoptosis of HCT-116 cells. With FC ≥ 2.0 and *p* value < 0.05 thresholds, a total of 240 lncRNAs, 131 circRNAs, 83 miRNAs, and 1415 mRNAs with significant differential expression were identified in the QUE-treated cells compared with the untreated cells. We found that several DEmRNAs and DEmiRNAs may be connected with CRC. However, the great majority of DElncRNAs and DEcircRNAs were not known, mainly due to rarely related research. In addition, 12 dysregulated ncRNAs and mRNAs identified were selected for qRT-PCR validation, and the results confirmed the sequencing analysis findings to some extent. On the basis of the KEGG analysis, two significantly enriched pathways, PI3K-Akt and Ras signaling pathway, were participated by all four RNAs. Previous studies have reported that these two cancer-related pathways may be the possible molecular mechanisms of QUE in anti-CRC (Kim et al., 2005; Psahoulia et al., 2007; Xavier et al., 2009; Yang et al., 2016). Our research results further confirmed this possibility, more deeply, comprehensively, and systematically.

Firstly, we focused on the differentially expressed coding genes. The top 20 DEmRNAs were regarded as the most important ones involved in mechanism of QUE acting on CRC. Among them, pioneering studies demonstrated that their dysregulation may result in the progression of CRC, such as AZGP1 (Ji et al., 2013; Chang et al., 2014; Xue et al., 2014b), APOBEC3G (Ding et al., 2011; Lan et al., 2014), BST2 (Mukai et al., 2017), TRIM29 (Jiang et al., 2013; Xu et al., 2016), and S100A4 (Dahlmann et al., 2014; Fei, 2017). Our results first suggested that MAGEA6, TNFSF12-TNFSF13, ANK1, PRR15L, BAG6, CD79B, KRT20, CTD-2410N18.5, RP11-385D13.1, HLA-DRB1, TTYH1, TNFRSF6B, PPP1R1B, SMIM5, and PTGDS may also exert their functions. The KEGG pathway analysis indicated that QUE-responsive gene alterations in HCT-116 cells were significantly enriched in the MAPK signaling pathway, a widely known cancer-related pathway. LRG1, which enriched in the MAPK pathway, has been strongly associated with worse overall survival for CRC, and may be considered as an independent prognostic indicator for CRC (Ladd et al., 2012; Zhang et al., 2016; Zhou et al., 2017; Zhang et al., 2018). In this study, LRG1 was proved to be down-regulated in QUE-treated HCT-116 cells compared to the untreated HCT-116 cells. These investigations indicated that LRG1 might be a potential drug target of QUE acting on CRC by regulating the MAPK signaling pathway.

Subsequently, the effects of QUE on ncRNAss, including miRNAs, lncRNAs, and circRNAs, were evaluated in the current study. Previous studies indicated that miR-338-3p dysregulation may contribute to the progression of CRC (Xue et al., 2014a). In our study, miR-338-3p was significantly up-regulated in HCT-116 cells treated with QUE, and was the top regulated ones. We suggested that miR-338-3p may be an important one participating in QUE’s anti-CRC mechanism. Additionally, four DEmiRNAs were referred as the most likely candidate miRNA associated with the mechanism of QUE. Among these, the expression of miR-320b, miR-320c, and miR-320d was significantly lower in CRC tissues than in normal tissues (Li et al., 2012). In our research, these three miRNAs were up-regulated in HCT-116 cells treated with QUE. These observations suggested that miR-320b, miR-320c, and miR-320d may play vital roles in anti-CRC mechanism of QUE. MiR-125b-2-3p, significantly down-regulated miRNA in CRC, was also pointed out as a novel diagnostic and prognostic biomarker in human CRC (Zhou et al., 2018). Our data showed that miR-125b-2-3p was up-regulated in QUE-treated HCT-116 cells. These studies suggested that miR-125b-2-3p may play an important role in the anti-CRC mechanism of QUE. Furthermore, some DEmiRNAs, such as novel_miR_873, novel_miR_710, novel_miR_20, and novel_miR_885, were also found to be significantly different between CRC cells with and without treatment of QUE, which suggested that these miRNAs play an important role in the anti-CRC mechanism of QUE. The relationships between these genes and CRC were firstly reported.

We noticed that a significant GO term of DElncRNAs and their target gene were related with DNA repair. This phenomenon is very illuminating, given the importance of DNA repair and damage in cancer (Mouw et al., 2017). Consistent with the results of GO analysis, KEGG pathway analysis also revealed that pathways associated with mismatch repair, nucleotide excision repair, and base excision repair were among the top regulated ones. From the result of the lncRNA–miRNA–mRNA network, we found that ENST00000313807 and ENST00000449307 were co-expressed with LRG1, which played important roles in anti-CRC mechanisms of QUE, through competitively binding with miR-5096. Additionally, MSTRG.13489.1, MSTRG.64442.1, MSTRG.128888.1, MSTRG.126191.1, ENST00000449307, ENST00000430883, ENST00000428222, MSTRG.71538.4, ENST00000434051, and MSTRG.211506.21 were identified to bind miR-338-3p competitively with its binding sites. None of these lncRNAs have been reported to be functional during CRC.

Increasing evidence indicate that circRNAs can influence miRNA activity as endogenous sponges and affect mRNA splicing and transcription by interacting with the Pol II complex in the nucleus (Tay et al., 2014; Xie et al., 2017). As many circRNAs failed to be allocated to functional modules, little public data about these circRNAs could be found. In this study, based on the constructed circRNA–miRNA–mRNA co-expression network, we observed that many circRNAs contained one or more miRNA binding sites. Thus, circRNA (8:93786223|93822563) was able to interact with LRG1, through competitively binding with miR-5096. This competitively binding mode was similar with ENST00000313807 and ENST00000449307. Therefore, further study was deserved to reveal the interaction relationships of circRNA (8:93786223|93822563)–miR-5096–LRG1 in QUE’s action mechanism.

Although altered ncRNAs and mRNAs were identified and their possible roles in anti-CRC mechanisms of QUE were investigated, several limitations should be considered in interpreting our findings. Firstly, a previous study reported that maximum plasma concentrations of QUE after the ingestion of 100 mg were lower than 10 µM (Graefe et al., 2001), and only one-tenth of the lower QUE concentration is used in this *in vitro* assays. Recently, the daily dose of QUE in a clinical study was up to 1250 mg for three consecutive days/week (Justice et al., 2019). Merging these results, one may speculate that those effective tissue levels could be attained. Thus, we selected relatively high concentrations of QUE to investigate its effects on the CRC cell line. In future studies, we could perform an analysis by exposing cells to lower QUE concentrations, aiming to investigate whether lower concentrations of QUE have anti-CRC efficacy and different impacts on gene expression profiles. Secondly, the analysis was only performed on HCT-116 cancer cells. Global ncRNA and mRNA changes in other CRC cell lines and CRC model animals treated with QUE should be also determined in further studies to more accurately reflect the anti-CRC mechanisms of QUE. Thirdly, RNA-sequence technology should be applied to unravel previously inaccessible transcriptome complexities, and because the functions of ncRNAs remain largely unknown, the comprehension of our data was not straightforward (Wang et al., 2018). Finally, further research should select more ncRNAs and mRNAs for sequencing data validation and observe whether the apoptotic process of HCT-116 cells will change by manipulating the expression of top nRNA and mRNA candidates. To solve this problem, further studies are now highly warranted.

## Conclusion

In summary, changes in the coding and non-coding transcriptomes of HCT-116 cells without and with QUE intervention were identified, and combining it with bioinformatics analysis may provide a better understanding of the potential roles of miRNA, lncRNA, circRNA, and mRNA in CRC and QUE treatment. To better reveal the mechanism of QUE-mediated apoptosis in HCT-116 cells, we now need to conduct further experiments through manipulating the action of the top potential candidates in this study. Moreover, the corresponding roles and molecular mechanisms of these ncRNA and mRNA need to be further explored.

## Data Availability

All the relevant data are contained within the manuscript.

## Author Contributions

BY contributed to the conception and the design of this study. ZZ, BL, and PX conducted the experiments. ZZ and BY drafted the main manuscript text. All the authors participated in the interpretation of results. All the authors have read and approved the final manuscript.

## Funding

This work was financially supported by the Hunan Science and Technology Department Project (No. 2014SK3039) and the implementation plan issued by the Office of the State Administration of Traditional Chinese Medicine for the fifth batch of national academic succession work for veteran Chinese medicine experts (2012, No. 40).

## Conflict of Interest Statement

The authors declare that the research was conducted in the absence of any commercial or financial relationships that could be construed as a potential conflict of interest.
